# Processing and Preservation of Aquatic Products

**DOI:** 10.3390/foods12102061

**Published:** 2023-05-19

**Authors:** Tao Yin, Liu Shi

**Affiliations:** 1 College of Food Science and Technology, Huazhong Agricultural University, Wuhan 430070, China; 2 Institute for Agricultural Products Processing and Nuclear—Agricultural Technology, Hubei Academy of Agricultural Science, Wuhan 430064, China; shiliu@hbaas.com

## 1. Introduction

Aquatic products represent an important food source; they include products such as fish, shrimp, shellfish, crab, and seaweed, and provide high-quality proteins, fatty acids, minerals, and other nutritional elements. Generally, raw aquatic materials must go through a production chain, including growth, harvesting, processing, storage, and circulation—from the waters to the dining table. Among these stages, processing is one of the most important. Through processing, product quality improvement, diversification, and comprehensive economic benefits can be achieved. Like processing, storage is also an important production stage. Extending the storage shelf life of aquatic products through preservation techniques can reduce loss, and also increases the convenience of product consumption. Using a cross-sectional consumer survey covering the main cities of the seven regions of Turkey, Güney et al. [1] applied an ordered probit model and descriptive statistics to analyze the effects of attitudes and socio-demographic characteristics (as independent variables) on fish consumption and purchase intention (as the dependent variables) in 421 participants. They found that taste, physical appearance, convenience, etc., have a significant positive relationship with the frequency of fish purchase and consumption. Therefore, selecting appropriate processing and preservation techniques to improve the quality of aquatic products and meet consumer needs represents the key to achieving enhanced economic value.

## 2. Processing of Aquatic Products

### 2.1. Raw Materials

Compared to raw food materials such as grains, wheat, and cattle, there is a much richer variety of aquatic products available for processing, including more than 2000 species. The qualities of a single species of fish, such as its nutritional composition, efficacy, and sensory properties, are influenced by environmental factors, size, season, gender, and other factors [2,3]. Snakehead fish (*Channa argus*), commonly known as black fish, are widely distributed in rivers, lakes, ponds, and swamps in China, Korea, Japan, Southeast Asia, India, and the Russian Far East. Snakehead fish are mainly farmed at high density or live in wild fish ponds. Ren et al. [2] prepared soups from farmed and wild snakehead fish and analyzed the nutritional composition of the soups. According to their results, there were no obvious differences in the nutritional composition or antioxidant activity between the soups. The protein content (1.90%) of the soup made from wild snakehead fish was relatively lower, but the total fatty acid (16.22 g/100 g), polyunsaturated fatty acid (7.17 g/100 g), and Zn (12.57 mg/kg) contents were significantly higher, suggesting that it may have better efficacy in promoting wound healing. Catfish are a significant commercial food commodity. Haque et al. [3] compared the proximate composition, retained water, and bacterial loads of two sizes of hybrid catfish (*Ictalurus furcatus × Ictalurus punctatus*) fillets. Additionally, they analyzed the impact of season on the differences in the raw materials. It was found that the water content (78.0 vs. 76.0%) was higher, and fat content (6.0% vs. 8.0%) was lower for smaller (50–150 g) fillets compared to larger fillets (150–450 g). Furthermore, higher levels of psychrotrophics (~4.2 vs. ~3.0) and total coliform (~3.4 vs. ~1.7) were observed in fillets in the warmer seasons (April–July) compared to the cold seasons (Feb–April). Therefore, understanding the differences in the raw materials of aquatic products is beneficial for ensuring quality control during processing, and ultimately, maintaining high end product quality.

The quality of crab and fish cultured in outdoor earthen ponds can be affected by numerous factors, such as the culture environment [4] and feeding [5]. For instance, compared with fresh water, fattening male Chinese mitten crabs (*eriocheir sinensis*) in saline and alkaline water led to numerous advantages, including improvements in carotenoid accumulation in freeze-dried carapaces, DHA, EPA, total free essential amino acids, total free amino acids, and total umami value in the hepatopancreas and muscle [4]. During transportation, fish experience a decrease in muscle quality due to the stress induced by various factors (e.g., ammonia nitrogen, collision, noise, and temperature). Peng et al. [5] temporarily raised Wuchang bream fish (*Megalobrama amblycephala*) for 48 h before transportation, and found that it significantly improved the muscle quality of the fish, including the water holding capacity and shear force. The reason for this may be that the intestinal emptying of the fish during the temporary respite process reduces ammonia nitrogen accumulation and fish stress during transportation. Improving the quality of raw materials before processing has positive significance.

### 2.2. Pretreatment

The initial stage of aquatic product processing, also known as pretreatment, generally includes grading, bleeding, cooling, scaling, head removal, visceral removal, etc. The main purpose of pretreatment is to remove non-edible portions such as the scales, viscera, head, and tail, which comprise up to 70% of the total body weight. In addition, this stage also includes maintaining the clean, white appearance of the fish meat and inhibiting microbial growth. Pretreatment is a crucial step in processing, and determines the quality, yield, and profits. Cutting comprises a set of crucial operations in fish pretreatment, whereby the whole fish is divided into smaller pieces or certain sections are cut off to produce fish products (e.g., fish fillets, steaks, surimi, slices, etc.). Traditionally, the cutting process was performed by humans using metal knives, which is labor-intensive. This process has two drawbacks. During cutting, a considerable amount of debris is created, which leads to a large amount of raw fish waste. Additionally, heavy metal food contamination is expected under an aqueous medium when a metal knife is used. In recent years, novel techniques (such as waterjet cutting, machine vision, and artificial intelligence) have been invented to overcome the drawbacks of metal blade cutting, and to achieve higher cutting efficiency and quality [6].

### 2.3. Aquatic Product Processing

Aquatic processing products include surimi and its products, frozen headed and gutted fish and fish fillets, fermented fish meat, dried squid and seaweed, canned fish meat, etc. Surimi is a concentrated myofibril protein, and is an intermediate raw material for processing surimi products. Washing is a key process in surimi processing. Water-soluble cathepsin, blood and fishy substances, as well as most fats can be removed through washing. The number of washes directly affects the quality and economic benefits of surimi. By comparing different numbers of washes (0, 1, 2, 3), Zhang et al. [7] found that the yield, types of fatty acids, redness (a*), total volatile basic nitrogen, and thiobarbituric acid-reactive substances of the surimi decreased, and the whiteness, pH, gel strength, and water retention increased, with increasing numbers of washes. They suggested that two washes was optimal, taking into account yield, water consumption, and surimi quality. The production of surimi using the traditional washing method has the disadvantages of bony structures, high levels of cathepsins, and a muddy off-odor occurring in the final product, the latter of which is mainly caused by geosmin (GEO) and 2-methylisoborneol (MIB). The use of a pH-shifting process not only significantly improves yield, but also effectively removes GEO and MIB. Guo et al. [8] studied the effects of acid and alkali extractions on the quality of surimi from silver carp (*Hypophthalmichthys molitrix*), and claimed that the alkali extraction method was a better alternative for making water-washed surimi from silver carp. However, currently, it is still difficult to achieve commercialization using the method of pH-shifting in surimi processing, which may be due to the difficulty in scaling up production and the high level of environmental pollution.

Surimi products are a type of deeply processed aquatic product with characteristics such as high protein, low fat, and an elastic texture. The processing of surimi products generally includes sequential processes such as thawing, cutting, chopping, the addition of ingredients, shaping, heating, and packaging. Adding functional ingredients such as starch, calcium ions, and transglutaminase can significantly improve the quality of surimi products. Sang et al. [9] added different proportions (0 to 4.5%) of calcium lactate to large yellow croaker (*Pseudosciaena crocea*) surimi and found that calcium lactate could significantly improve its gelation properties, including its gel strength, whiteness, cooking loss and water holding capacity. The mechanism of calcium lactate enhancing surimi gelation was found to be related to the promotion of myosin stretching by divalent calcium ions and the formation of salt bridges between adjacent negatively charged proteins. The authors claimed that 1.5% was the optimal amount of calcium lactate.

Fermented aquatic products are also an important type of aquatic processing products. In the processing of aquatic products, it is generally necessary to use methods such as ozone water washing and to operate at low temperatures to reduce microorganisms, in order to prevent raw materials from spoiling and deteriorating. Contrary to other aquatic products, fermented aquatic products utilize the activity of microorganisms to partially decompose aquatic proteins and partially oxidize fats, producing unique flavors. Due to differences in raw materials and fermentation processes, various fermented aquatic products with local characteristics are formed, such as “Zhayu”, which is a popular fish product in central provinces of China. Compared with natural fermentation, inoculated microbial fermentation has the significant advantages of shortening production cycles and improving product quality stability. An et al. [10] found that inoculating lactic acid bacteria could increase the crude fat and protein content of “Zhayu”, reduce the fishy smell, and make the product tender and soft. According to their research results, the samples prepared with 10^9^ cfu/100 g lactic acid bacteria presented the best overall qualities. In addition, they also reported significant differences in the quality of “Zhayu” produced via inoculation with three different types of lactic acid bacteria. “Zhayu” fermented with *L. plantarum* and *P. acidilactici* showed the strongest sourness, while the samples prepared with *P. pentosaceus* showed the strongest umami taste, as evidenced by the fact that they had the highest Asp (25.1 mg/100 g) and Glu (67.8 mg/100 g) content.

Besides fish, seaweed is also widely available and of great commercial importance. At present, seaweed is mainly used for extracting polysaccharides, or dried for consumption in dishes and casual snacks. In a recent study, it was stated that powdered seaweed (*Ulva intestinalis*) from coastal areas of Bangladesh is incorporated into cookies to enrich them in unsaturated fatty acids [11].

## 3. Preservation of Aquatic Products

### 3.1. Preserving Agents

The muscle tissue of fish and other aquatic products is soft and tender, with high water content. The enzymes in a fish’s body exhibit strong activity at room temperature. After the fish dies, these enzymes quickly decompose the fish protein into a large number of low-molecular metabolites and free amino acids, which become nutrients for bacteria. Therefore, aquatic products are generally prone to spoilage. Thanks to the advancement of preservation and rapid detection technologies (such as employing the peroxide activity of platinum cubes as an indicator of fish freshness [12]), the shelf life of aquatic products can be extended and safety hazards can be reduced. Adding a preserving agent is an effective means of extending the shelf life of aquatic products. In recent years, the use of weak acids [13,14,15], essential oils [16,17], and epigallocatechin gallate (EGCG) [18] as aquatic preserving agents has become a research hotspot.

Zhan et al. [13] applied sous vide (SV) cooking treatments to half-shell scallop (*Chlamys farreri*), and compared its physicochemical qualities and the volatility of its flavor with a control (cooked at 100 °C for 10 min) during 30 d of chilled storage. Upon monitoring the volatile basic nitrogen (TVBN), pH, texture, malondialdehyde (MDA) content, and myofibrillar protein (MP) extraction rate of the scallop samples, they reported that the SV cooking treatments effectively maintained acceptable and stable physicochemical qualities during storage. The effects of different acids on the quality, nutrition, and sensory quality of aquatic products vary. For example, Baltic herring (*Clupee harengus membras*) pickled in acetic acid and malic acid showed lower fat content during storage compared with those pickled in citric, lactic, and tartaric acids [14]. Replacing acetic acid with other weak acids resulted in pickled and marinated Baltic herring with novel and milder sensory profiles. There is a synergistic effect between weak acids and other physical preservation technologies. Lactic acid in combination with high hydrostatic pressure effectively inhibited microbiological growth and physicochemical changes (pH, sensory evaluation, flush, and texture) of large-mouth bass fillets [15].

Essential oils (EO) are important natural plant extracts and have attracted much interest from scientists for their antibacterial and antioxidant properties. By comparing four essential oils (oregano essential oil (OEO), tea tree essential oil (TTEO), and wild orange essential oil (WOEO)), Qian et al. [16] reported that OEO displayed the highest antimicrobial effect in Pacific white shrimp (*Litopenaeus vannamei*) during cold storage. Additionally, they found that the combination of OEO and CLEO had a synergistic effect, and displayed the highest efficacy in preventing melanosis, bacterial growth, and protein hydrolysis in shrimp. In order to overcome the volatile nature of essential oils, Qiu et al. [17] processed microcapsules with porous starch as an adsorbed substrate to store SOEO (PS/SOEO), sodium alginate (SA), and chitosans (CMCSs) as shell materials, in order to delay the volatilization of SOEO. They found that CMCS and SA improved the slow reducing ability of SOEO microcapsules, and the shelf life of crawfish (*Procambarus clarkii*) could be extended to 6 days by SOEO microcapsules (1/10 g, SOEO microcapsules/crowfish) at room temperature.

Like essential oils, tea polyphenols are an emerging food preserving agent for aquatic products due to their vital antioxidant and bacteriostatic properties. According to a study by Tian et al. [18], besides maintaining texture and gel strength, the addition of EGCG can inhibit microbial growth and the formation of off-odor compounds such as total volatile basic nitrogen (TVB-N) and malondialdehyde (MDA). They recommended that EGCG at a concentration of 0.01–0.02% could effectively preserve surimi gels during freeze–thaw cycles.

### 3.2. Non-Thermal Processing

In recent years, non-thermal processing has been increasingly applied to the preservation of aquatic products. Compared with thermal sterilization such as pasteurization, it can better preserve the nutrition, texture, and flavor of aquatic products. Common non-thermal processing methods for aquatic products include ultrasound [19], high-voltage atmospheric cold plasma [20], electron-beam irradiation [21], high hydrostatic pressure [15], etc.

Ling et al. [19] investigated ozone water and ultrasound cleaning in the storage of crayfish through microbial viable counts and 16S rRNA gene sequencing. They reported that the ozone water in combination with ultrasound cleaning could significantly reduce the total viable count, psychrophilic viable count, mesophilic viable count, pseudomonas, hydrogen sulfide-producing bacteria, molds, and yeasts. With the extension of high-voltage atmospheric cold plasma treatment (0 to 300 s), the number of colonies on the surface of tilapia (*Oreochromis mossambicus*) fillets inoculated with *Salmonella enterica serovar enteritid* is (*S. enteritis*) and/or *Listeria monocytogenes* (*L. monocytogenes*) gradually decreased [20]. However, the pH, b* value, elasticity, chewiness, thiol value, and TVB-N value were not significantly different. Electron-beam irradiation treatment with a dose of 4 kGy was able to retain the muscle water content and preserve their quality of silver carp (*Hypophthalmichthys molitrix*) chunks stored at 4 °C [21]. Additionally, high hydrostatic pressure treatment at 400 MPa effectively inhibited microbiological growth and physiochemical changes in large-mouth bass fillets [15]. The mechanism by which non-thermal processing exerts its preservation effect is generally related to its damage to microbial cell membranes and enzyme/protein structures. Briefly, non-thermal processing can be used as a cleaning strategy to control the microbiological quality of aquatic products, and has much lighter effects on their quality.

### 3.3. Packaging

Recently, in addition to air packaging and vacuum packaging, modified atmosphere packaging (MAP) has also been utilized in aquatic preservation. Regarding MAP, O_2_, CO_2_, and N_2_ gas are mixed into high-barrier-performance packaging material to inhibit microbial growth, enzymatic reactions, and lipid oxidation. In a recent study, Zhang et al. reported that MAP with a 70% CO_2_/30% N_2_ gas ratio was optimized to inhibit the quality deterioration of golden pompano (*Trachinotus ovatus*) fillets [22].

Aquatic products are an important source of food for humans. The processing and preservation techniques for aquatic products are constantly improving, with the aim of improving nutrition, taste, and convenience. In the production process of aquatic products, enterprises are also increasingly emphasizing energy conservation, loss reduction, and environmental protection. It can be foreseen that these techniques, which are still in their experimental and pilot stages, will play an increasingly important role in future industrial production. New processing and preservation techniques will also continue to emerge.

In this editorial, with regard to aquatic product processing and preservation, we introduce methods of raw material quality improvement (using fattening techniques in saline and alkaline water and respite as examples), pretreatments (using cutting as examples), and new techniques for processing aquatic products (using surimi and its products, fermented products, and seaweed products as examples). In addition, we introduce new technological advancements in the preservation of aquatic products from the perspectives of preserving agents (using weak acids, essential oils, and EGCG as examples), non-thermal processing (using ultrasound, high-voltage atmospheric cold plasma, electron-beam irradiation, and high hydrostatic pressure as examples), and packaging (using MAP as an example). This editorial is suitable for undergraduate and graduate students majoring in food science and technology.

We are grateful to the contributing authors of this volume for their efforts and excellent work. It is hoped that readers of this text will find it useful and direct constructive comments regarding its content (as well as printing errors) to our attention.

## Data Availability

Not applicable.
